# (4,7,13,16,21,24-Hexaoxa-1,10-di­aza­bicyclo­[8.8.8]hexa­cosa­ne)sodium iodide–1,1,2,2,tetra­fluoro-1,2-diiodo­ethane (2/3)

**DOI:** 10.1107/S1600536813016085

**Published:** 2013-06-15

**Authors:** Gabriella Cavallo, Pierangelo Metrangolo, Tullio Pilati, Giuseppe Resnati, Maurizio Ursini, Giancarlo Terraneo

**Affiliations:** a NFMLab, Department of Chemistry, Materials and Chemical Engineering, "Giulio Natta", Politecnico di Milano, Via Mancinelli, 7, I-20131 Milano, Italy

## Abstract

The title complex (CX1), [Na(C_18_H_36_N_2_O_6_)]I·1.5C_2_F_4_I_2_, is a three-component adduct containing a [2.2.2]-cryptand, sodium iodide and 1,1,2,2-tetra­fluoro-1,2-di­iodo­ethane. The di­iodo­ethane works as a bidentate halogen-bonding (XB) donor, the [2.2.2]-cryptand chelates the sodium cation, and the iodide counter-ion acts as a tridentate XB acceptor. A (6,3) network is formed in which iodide anions are the nodes and halocarbons the sides. The network symmetry is *C*_3i_ and the I⋯I^−^ XB distance is 3.4492 (5) Å. This network is strongly deformed and wrinkled. It forms a layer 9.6686 (18) Å high and the inter-layer distance is 4.4889 (10) Å. The cations, inter­acting with each other *via* weak O⋯H hydrogen bonds, are confined between two anionic layers and also form a (6,3) net. The structure of CX1 is closely related to that of the KI homologue (CX2). The 1,1,2,2,-tetrafluoro-1,2-diiodoethane molecule is rotationally disordered around the I⋯I axis, resulting in an 1:1 disorder of the C_2_F_4_ moiety.

## Related literature

For other K2.2.2./salt/haloperfluoro­carbon complexes, see: Fox *et al.* (2004 ▶); Metrangolo *et al.* (2004 ▶); Liantonio *et al.* (2003 ▶, 2006 ▶).
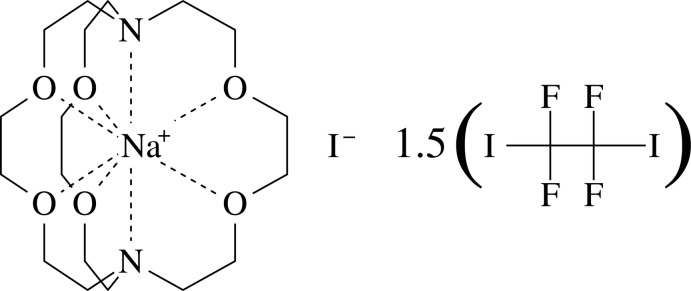


## Experimental

### 

#### Crystal data

[Na(C_18_H_36_N_2_O_6_)]I^−^·1.5C_2_F_4_I_2_
*M* *_r_* = 1057.11
Trigonal, 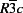

*a* = 11.634 (2) Å
*c* = 84.945 (15) Å
*V* = 9957 (4) Å^3^
*Z* = 12
Mo *K*α radiation
μ = 3.84 mm^−1^
*T* = 93 K
0.28 × 0.25 × 0.03 mm

#### Data collection

Bruker APEXII CCD diffractometer
Absorption correction: multi-scan (*SADABS*; Bruker, 2008 ▶) *T*_min_ = 0.676, *T*_max_ = 1.000
47282 measured reflections
2994 independent reflections
2604 reflections with *I* > 2σ(*I*)
*R*_int_ = 0.035

#### Refinement

*R*[*F* ^2^ > 2σ(*F* ^2^)] = 0.032
*wR*(*F* ^2^) = 0.076
*S* = 1.07
2994 reflections
148 parameters
44 restraints
H-atom parameters constrained
Δρ_max_ = 1.64 e Å^−3^
Δρ_min_ = −0.58 e Å^−3^

### 

Data collection: *APEX2* (Bruker, 2008 ▶); cell refinement: *SAINT* (Bruker, 2008 ▶); data reduction: *SAINT*; program(s) used to solve structure: *SIR2002* (Burla *et al.*, 2003 ▶); program(s) used to refine structure: *SHELXL2012* (Sheldrick, 2008) ▶; molecular graphics: *ORTEP-3 for Windows* (Farrugia, 2012 ▶) and *Mercury* (Macrae *et al.*, 2006 ▶); software used to prepare material for publication: *SHELXL2012*.

## Supplementary Material

Crystal structure: contains datablock(s) global, I. DOI: 10.1107/S1600536813016085/kj2225sup1.cif

Structure factors: contains datablock(s) I. DOI: 10.1107/S1600536813016085/kj2225Isup2.hkl

Additional supplementary materials:  crystallographic information; 3D view; checkCIF report

## Figures and Tables

**Table 1:** Some parameters (Å, Å^3^) of the anionic layer and of the cation in the structures CX1 and CX2

	CX1	CX2
Hole side^1^	11.634 (2)	11.7478 (15)
Layer height^2^	9.6686 (18)	9.6380 (13)
*h* ^3^	4.4889 (10)	4.5343 (7)
*V* ^3^	303.79 (7)	312.89 (6)
*M* ^+^—O1	2.460 (2)	2.6650 (12)
*M* ^+^—O2	2.692 (2)	2.7737 (13)
*M* ^+^—N1	2.744 (5)	2.941 (2)
*M* ^+^—N2	3.271 (5)	2.985 (3)

Notes: (1) Distance between the nearest iodide anions on the same side of the anionic layer, equal to the cell parameter *a*; (2) distance between the planes through the iodide anions on the opposite sides of the anionic layer; (3) *h* = distance between the nearest planes through iodide anions of contiguous layers. (4) *V* = *a* ^2^ *h*/2, volume of the trigonal prism whose vertices are the three iodide anions on a layer and the same faced on the contiguous one.

**Table 2:** Halogen and hydrogen bonds (Å, °) in CX1 and CX2 In CX2, the cell origin and the atom numbering are different, so that atom labels and symmetry code refer only to CX1; for CX2 the reported values refer to the equivalent atoms and values.

*X*⋯*Y*—C	CX1 *X*⋯*Y*	CX1 C—*X*⋯*Y*	CX2 *X*⋯*Y*	CX2 C—*X*⋯*Y*
I2⋯I1—C7	3.4492 (5)	175.99 (17)	3.4492 (5)	176.30 (16)
I2⋯I1—C8^i^	3.4492 (5)	168.30 (16)	3.4492 (5)	166.40 (16)
O1⋯(H3*B*—C3)^ii^	2.63	147.9	2.60	147.6

Symmetry codes: (i) 
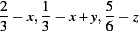
; (ii) 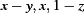.

**Fig. 1.**
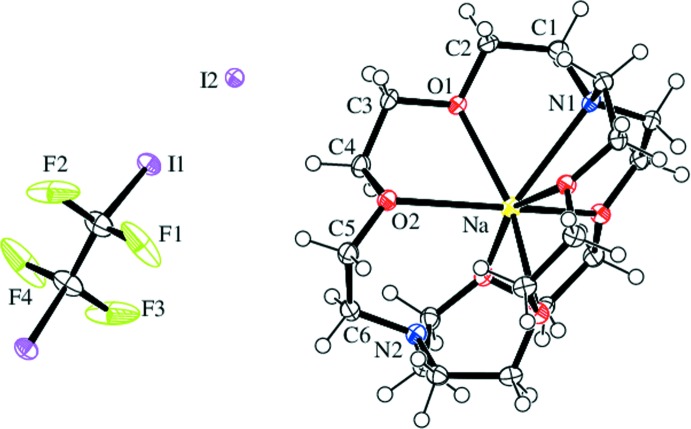
The three components of CX1, with numbering scheme of the indepent atoms. The disordered atoms of DIPFA2, generated by the twofold axis are omitted for clarity. Probability level at 50%.

**Fig. 2.**
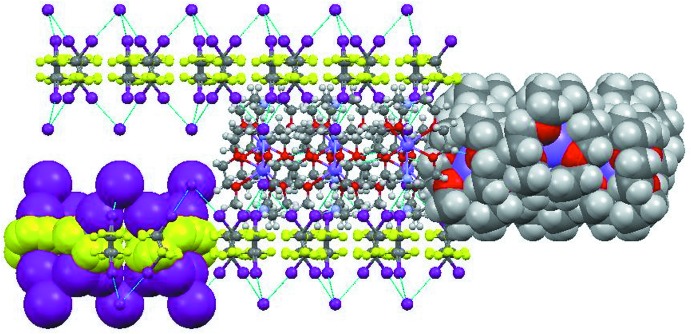
A layer of cations and two layers of anions, are shown along the a* axis, only partial overposition is adopted for sake of clarity. One hexagonal ring of supramolecular cations and of supramolecular anions are the topologic units of the layers and are shown in spacefilling style.

**Fig. 3.**
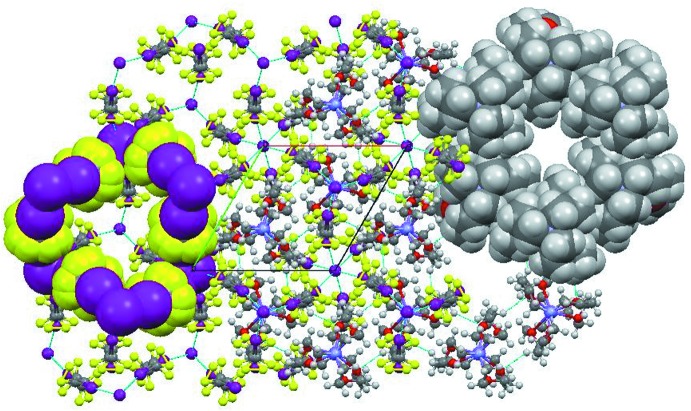
The same molecular assembly as shown in Figure 2, projected down the c axis

## supplementary crystallographic information

### Comment

(4,7,13,16,21,24-hexaoxa-1,10-diazabicyclo(8.8.8)hexacosane (K2.2.2) is one of the most popular cryptands in supramolecular chemistry and crystal engineering. Our group has used this [2.2.2] cryptand to generate naked halide anions from their alkali and alkali earth salts and to promote the formation of halogen bonding (XB) with diiodoperfluoroalkanes (DIPFA_n_, where n is the alkyl chain length). Different structures were obtained as a function of the cation and of the haloalkane length. For instance, in the complex with BaI_2_ and DIPFA_2_ the ratios K2.2.2/BaI_2_/DIPFA_2_ are 1:1:1, iodide anions function as monodentate XB acceptors and form the trimer I^-^···DIPFA_2_···I^-^ (Fox, *et al.*, 2004). This is probably related to the fact that the iodide anions are hydrogen bonded to a water molecule and the resulting decrease of electron density on the anion may limit the number of XB's it gives rise to. The K2.2.2/BaI_2_/DIPFA_8_ adduct presents a quite different stoichiometry and interaction pattern (Metrangolo, *et al.*, 2004). Here, the ratios among the three component are 1:1:3 and an infinite comb-like supramolecular anion is formed in which iodide anions in the main chain and in the prongs function as tridentate and bidentate XB acceptors, respectively. Here too the cryptand does not saturate the cation coordination sphere and two methanol molecules are bound to barium. In the K2.2.2/KI/DIPFA_n_ adducts (n = 2,6 (Liantonio, *et al.*, 2006) and n=4,8 (Liantonio, *et al.*, 2003) the ratios among the three components is 2:2:3. The cryptand completes the coordination sphere of K^+^ cation and no water or alcohol molecules are present in the crystals. The iodide anions are free to function as tridentate XB acceptors and unlimited (6,3) anionic networks are formed in all four cases. This net is not planar but strongly wrinkled as the (C—I)_3_···I^-^ group is pyramidal. The six iodide nodes are the vertices of a trigonal anti-prism whose dimension can be fully described by the mean distance between two iodide anions on the same side of the layer, and by the distance between the planes through the iodide nodes on the two layer sides. The hole dimension increases with n and for n=6,8 it is so large that the cation cannot fulfill the voids and three different (6,3) nets interpenetrate to give an intriguing borromean system (Liantonio, *et al.*, 2006). In all four structures, two layers are faced vertex to vertex, hole to hole, as two egg trays where the cations are hosted. In the K2.2.2/NaI/DIPFA_2_ adducts (CX1) described here, I^-^ anions are tridentate XB acceptors, DIPFA_2_ are bidentate XB donors and a (6,3) net is formed which is closely similar to that of the KI analogue (CX2). Figure 1 shows the molecular geometry, with the numbering scheme. The Na^+^ cation is small relative to the cryptand cavity and is therefore not exactly in the middle of the [2.2.2] cryptand cavity, as was the case for K^+^ in CX2. As a consequence, the two independent Na^+^—N distances are very different. Table 1 reports some geometric details of the supramolecular anion hosting cavity and of the supramolecular cation dimensions in CX1 and CX2. Table 2 shows the halogen and hydrogen bonds of CX1. The 'egg tray' here is too small to isolate completely the 'eggs', namely the supramolecular cations, which are linked to each other by a couple of symmetry equivalent weak hydrogen bonds between the methylene hydrogen atom and ether oxygen forming a layer with the same topology of the anion network. Both the anion and cation layers are shown in Figure 2 and 3.

### Experimental

The complex was prepared in two steps. Equimolar amounts of [2.2.2] cryptand and NaI in ethanol solution were mixed and refluxed for 5 min. After cooling, the solution was added to a chloroform solution of DIPFA_2_ (1.5 equivalents). A glass vial containg the resulting mixture was put in a wide mouth flask containing vaseline oil. Vapour exchange at room temperature afforded colourless, thin, hexagonal crystals of good quality after a few days.

### Refinement

The tetrafluorodiiodoethane molecule was rotationally disordered. The split model was refined with restraints on geometric parameters and ADPs. The rotation of this molecule around the I···I axis, was so large that *SHELXL* suggested a second splitting of two F atoms. We considered this suggestion not useful and even dangerous to refinement stability in view of the high correlations between split atoms parameters (already up to 0.87). Hydrogen atoms were positioned geometrically and refined using a riding model, with C—H = 0.95–0.99 Å and with *U*_iso_(H) = 1.2 times *U*_eq_(C).

### Crystal data

**Table:**

[Na(C_18_H_36_N_2_O_6_)]I^−^·1.5C_2_F_4_I_2_	*D*_x_ = 2.116 Mg m^−^^3^
*M**_r_* = 1057.11	Mo *K*α radiation, λ = 0.71073 Å
Trigonal, *R*3*c*	Cell parameters from 20222 reflections
*a* = 11.634 (2) Å	θ = 2.2–29.8°
*c* = 84.945 (15) Å	µ = 3.84 mm^−^^1^
*V* = 9957 (4) Å^3^	*T* = 93 K
*Z* = 12	Hexagonal table, colourless
*F*(000) = 6012	0.28 × 0.25 × 0.03 mm

### Data collection

**Table:**

Bruker APEXII CCD diffractometer	2604 reflections with *I* > 2σ(*I*)
Radiation source: sealed tube	*R*_int_ = 0.035
φ and ω scans	θ_max_ = 30.0°, θ_min_ = 1.4°
Absorption correction: multi-scan (*SADABS*; Bruker, 2008)	*h* = −15→15
*T*_min_ = 0.676, *T*_max_ = 1.000	*k* = −15→15
47282 measured reflections	*l* = −114→114
2994 independent reflections	

### Refinement

**Table:**

Refinement on *F*^2^	Primary atom site location: structure-invariant direct methods
Least-squares matrix: full	Secondary atom site location: difference Fourier map
*R*[*F*^2^ > 2σ(*F*^2^)] = 0.032	Hydrogen site location: inferred from neighbouring sites
*wR*(*F*^2^) = 0.076	H-atom parameters constrained
*S* = 1.07	*w* = 1/[σ^2^(*F*_o_^2^) + (0.040*P*)^2^] where *P* = (*F*_o_^2^ + 2*F*_c_^2^)/3
2994 reflections	(Δ/σ)_max_ = 0.001
148 parameters	Δρ_max_ = 1.64 e Å^−^^3^
44 restraints	Δρ_min_ = −0.58 e Å^−^^3^

### Special details

**Table:**

Experimental. OXFORD low temperature device.
Geometry. All e.s.d.'s (except the e.s.d. in the dihedral angle between two l.s. planes) are estimated using the full covariance matrix. The cell e.s.d.'s are taken into account individually in the estimation of e.s.d.'s in distances, angles and torsion angles; correlations between e.s.d.'s in cell parameters are only used when they are defined by crystal symmetry. An approximate (isotropic) treatment of cell e.s.d.'s is used for estimating e.s.d.'s involving l.s. planes.
Refinement. The tetrafluorodiiodoethane molecule was rotationally disordered. The split model was refined with restraints on geometric parameters and ADPs. The rotation of this molecule around the *I*···*I* axis, was so large that *SHELXL* suggested a second splitting of two *F* atoms. We considered not useful and even dangerous the suggestion, because the largest correlations between split atoms parameters, already high (<0.87), would be larger.

### Fractional atomic coordinates and isotropic or equivalent isotropic displacement parameters (Å^2^)

**Table:**

	*x*	*y*	*z*	*U*_iso_*/*U*_eq_	Occ. (<1)
I2	0.0000	0.0000	0.47358 (2)	0.01498 (10)	
I1	0.22447 (2)	0.05878 (2)	0.44381 (2)	0.02001 (9)	
C7	0.3618 (9)	0.1058 (7)	0.42456 (12)	0.029 (2)	0.5
F1	0.4634 (7)	0.2274 (9)	0.42658 (10)	0.065 (3)	0.5
F2	0.4115 (9)	0.0252 (9)	0.42395 (11)	0.080 (3)	0.5
C8	0.3016 (8)	0.1016 (6)	0.40861 (12)	0.028 (2)	0.5
F3	0.2439 (9)	0.1750 (10)	0.40947 (12)	0.074 (3)	0.5
F4	0.2054 (7)	−0.0222 (8)	0.40618 (10)	0.083 (4)	0.5
Na	0.3333	0.6667	0.48841 (2)	0.0176 (4)	
N1	0.3333	0.6667	0.52071 (5)	0.0147 (9)	
C1	0.2462 (3)	0.5297 (3)	0.52603 (3)	0.0167 (6)	
H1A	0.1525	0.5071	0.5248	0.020*	
H1B	0.2622	0.5232	0.5374	0.020*	
C2	0.2684 (3)	0.4311 (3)	0.51696 (4)	0.0174 (6)	
H2A	0.3585	0.4461	0.5191	0.021*	
H2B	0.2029	0.3397	0.5202	0.021*	
O1	0.2542 (2)	0.44777 (19)	0.50055 (2)	0.0159 (4)	
C3	0.2593 (3)	0.3468 (3)	0.49158 (3)	0.0186 (6)	
H3A	0.1802	0.2595	0.4938	0.022*	
H3B	0.3394	0.3422	0.4944	0.022*	
C4	0.2632 (3)	0.3789 (3)	0.47452 (4)	0.0186 (6)	
H4A	0.2602	0.3067	0.4680	0.022*	
H4B	0.1857	0.3884	0.4718	0.022*	
O2	0.3838 (2)	0.5009 (2)	0.47155 (3)	0.0180 (5)	
C5	0.4235 (3)	0.5180 (3)	0.45540 (4)	0.0198 (6)	
H5A	0.4210	0.4363	0.4516	0.024*	
H5B	0.5162	0.5918	0.4546	0.024*	
C6	0.3357 (3)	0.5475 (3)	0.44487 (3)	0.0191 (6)	
H6A	0.3687	0.5597	0.4339	0.023*	
H6B	0.2442	0.4708	0.4450	0.023*	
N2	0.3333	0.6667	0.44990 (5)	0.0173 (9)	

### Atomic displacement parameters (Å^2^)

**Table:**

	*U*^11^	*U*^22^	*U*^33^	*U*^12^	*U*^13^	*U*^23^
I2	0.01522 (13)	0.01522 (13)	0.01451 (17)	0.00761 (6)	0.000	0.000
I1	0.01636 (13)	0.02578 (14)	0.01665 (12)	0.00961 (9)	0.00183 (7)	0.00086 (8)
C7	0.028 (5)	0.051 (6)	0.021 (5)	0.030 (5)	0.005 (4)	0.002 (4)
F1	0.025 (3)	0.080 (6)	0.025 (3)	−0.022 (4)	0.007 (2)	−0.019 (5)
F2	0.114 (8)	0.145 (7)	0.058 (6)	0.122 (7)	0.058 (5)	0.066 (5)
C8	0.017 (5)	0.048 (6)	0.020 (5)	0.017 (4)	0.004 (4)	0.003 (4)
F3	0.086 (7)	0.137 (7)	0.060 (6)	0.101 (6)	0.046 (5)	0.063 (6)
F4	0.033 (4)	0.089 (7)	0.028 (3)	−0.043 (4)	0.009 (3)	−0.022 (5)
Na	0.0162 (7)	0.0162 (7)	0.0204 (10)	0.0081 (3)	0.000	0.000
N1	0.0115 (13)	0.0115 (13)	0.021 (2)	0.0057 (6)	0.000	0.000
C1	0.0148 (14)	0.0158 (15)	0.0172 (14)	0.0058 (13)	0.0007 (11)	0.0012 (11)
C2	0.0184 (16)	0.0147 (15)	0.0184 (15)	0.0078 (13)	−0.0009 (12)	0.0023 (11)
O1	0.0194 (11)	0.0140 (11)	0.0163 (10)	0.0099 (9)	0.0001 (8)	0.0004 (8)
C3	0.0219 (16)	0.0120 (15)	0.0211 (15)	0.0079 (13)	0.0002 (12)	−0.0011 (11)
C4	0.0167 (15)	0.0135 (15)	0.0221 (15)	0.0049 (13)	0.0016 (12)	−0.0015 (12)
O2	0.0182 (11)	0.0143 (11)	0.0185 (11)	0.0058 (9)	0.0000 (8)	−0.0003 (8)
C5	0.0192 (16)	0.0188 (16)	0.0207 (15)	0.0091 (13)	0.0038 (13)	0.0002 (12)
C6	0.0197 (16)	0.0195 (16)	0.0168 (14)	0.0089 (13)	0.0001 (12)	−0.0016 (12)
N2	0.0167 (14)	0.0167 (14)	0.018 (2)	0.0084 (7)	0.000	0.000

### Geometric parameters (Å, º)

**Table:**

I1—C8^i^	2.153 (11)	O1—C3	1.427 (4)
I1—C7	2.156 (11)	C3—C4	1.492 (4)
C7—F1	1.325 (5)	C3—H3A	0.9900
C7—F2	1.327 (5)	C3—H3B	0.9900
C7—C8	1.514 (7)	C4—O2	1.434 (4)
C8—F4	1.325 (5)	C4—H4A	0.9900
C8—F3	1.327 (5)	C4—H4B	0.9900
N1—C1^ii^	1.468 (3)	O2—C5	1.430 (4)
N1—C1	1.468 (3)	C5—C6	1.520 (4)
N1—C1^iii^	1.468 (3)	C5—H5A	0.9900
C1—C2	1.508 (4)	C5—H5B	0.9900
C1—H1A	0.9900	C6—N2	1.464 (3)
C1—H1B	0.9900	C6—H6A	0.9900
C2—O1	1.428 (4)	C6—H6B	0.9900
C2—H2A	0.9900	N2—C6^iii^	1.464 (3)
C2—H2B	0.9900	N2—C6^ii^	1.464 (4)
			
F1—C7—F2	106.7 (7)	O1—C3—C4	108.7 (2)
F1—C7—C8	107.6 (5)	O1—C3—H3A	109.9
F2—C7—C8	107.4 (5)	C4—C3—H3A	109.9
F1—C7—I1	109.0 (5)	O1—C3—H3B	109.9
F2—C7—I1	112.3 (6)	C4—C3—H3B	109.9
C8—C7—I1	113.5 (4)	H3A—C3—H3B	108.3
F4—C8—F3	106.5 (7)	O2—C4—C3	108.1 (2)
F4—C8—C7	107.5 (5)	O2—C4—H4A	110.1
F3—C8—C7	107.7 (5)	C3—C4—H4A	110.1
F1^i^—C8—I1^i^	118.8 (9)	O2—C4—H4B	110.1
F4—C8—I1^i^	110.4 (6)	C3—C4—H4B	110.1
F3—C8—I1^i^	110.5 (6)	H4A—C4—H4B	108.4
C7—C8—I1^i^	114.0 (4)	C5—O2—C4	113.2 (2)
C1^ii^—N1—C1	110.97 (18)	O2—C5—C6	112.9 (3)
C1^ii^—N1—C1^iii^	110.97 (18)	O2—C5—H5A	109.0
C1—N1—C1^iii^	110.97 (18)	C6—C5—H5A	109.0
N1—C1—C2	112.3 (2)	O2—C5—H5B	109.0
N1—C1—H1A	109.1	C6—C5—H5B	109.0
C2—C1—H1A	109.1	H5A—C5—H5B	107.8
N1—C1—H1B	109.1	N2—C6—C5	112.0 (3)
C2—C1—H1B	109.1	N2—C6—H6A	109.2
H1A—C1—H1B	107.9	C5—C6—H6A	109.2
O1—C2—C1	108.6 (2)	N2—C6—H6B	109.2
O1—C2—H2A	110.0	C5—C6—H6B	109.2
C1—C2—H2A	110.0	H6A—C6—H6B	107.9
O1—C2—H2B	110.0	C6^iii^—N2—C6^ii^	111.84 (19)
C1—C2—H2B	110.0	C6^iii^—N2—C6	111.84 (19)
H2A—C2—H2B	108.3	C6^ii^—N2—C6	111.84 (18)
C3—O1—C2	110.8 (2)		
			
F1—C7—C8—F4	176.1 (9)	C1^iii^—N1—C1—C2	162.6 (3)
F2—C7—C8—F4	61.5 (8)	N1—C1—C2—O1	−54.8 (3)
I1—C7—C8—F4	−63.3 (8)	C1—C2—O1—C3	−173.3 (2)
F1—C7—C8—F3	−69.6 (8)	C2—O1—C3—C4	−172.2 (2)
F2—C7—C8—F3	175.9 (7)	O1—C3—C4—O2	63.8 (3)
I1—C7—C8—F3	51.1 (7)	C3—C4—O2—C5	158.4 (2)
F1—C7—C8—I1^i^	53.4 (8)	C4—O2—C5—C6	71.9 (3)
F2—C7—C8—I1^i^	−61.2 (7)	O2—C5—C6—N2	58.0 (3)
I1—C7—C8—I1^i^	174.0 (2)	C5—C6—N2—C6^iii^	−154.9 (3)
C1^ii^—N1—C1—C2	−73.6 (4)	C5—C6—N2—C6^ii^	78.7 (4)

Symmetry codes: (i) −*x*+2/3, −*x*+*y*+1/3, −*z*+5/6; (ii) −*y*+1, *x*−*y*+1, *z*; (iii) −*x*+*y*, −*x*+1, *z*.

### Some parameters (Å, Å^3^) of the anionic layer and of the cation in the structures CX1 and CX2.

**Table:**

	CX1	CX2
Hole side^1^	11.634 (2)	11.7478 (15)
Layer height^2^	9.6686 (18)	9.6380 (13)
*h*^3^	4.4889 (10)	4.5343 (7)
*V*^3^	303.79 (7)	312.89 (6)
*M*^+^—O1	2.460 (2)	2.6650 (12)
*M*^+^—O2	2.692 (2)	2.7737 (13)
*M*^+^—N1	2.744 (5)	2.941 (2)
*M*^+^—N2	3.271 (5)	2.985 (3)

Notes: (1) Distance between the nearest iodide anions on the same side of the anionic layer, equal to the cell parameter *a*; (2) distance between the planes through the iodide anions on the opposite sides of the anionic layer; (3) *h* = distance between the nearest planes through iodide anions of contiguous layers. (4) *V* = *a*^2^*h*/2, volume of the trigonal prism whose vertices are the three iodide anions on a layer and the same faced on the contiguous one.

### Halogen and hydrogen bonds (Å, °) in CX1 and CX2.

In CX2, the cell origin and the atom numbering are different, so that atom labels and symmetry code refer only to CX1; for CX2 the reported values refer to the equivalent atoms and values.

**Table:**

*X*···*Y*—C	CX1 *X*···*Y*	CX1 C—*X*···*Y*	CX2 *X*···*Y*	CX2 C—*X*···*Y*
I2···I1—C7	3.4492 (5)	175.99 (17)	3.4492 (5)	176.30 (16)
I2···I1—C8^i^	3.4492 (5)	168.30 (16)	3.4492 (5)	166.40 (16)
O1···(H3B—C3)^ii^	2.63	147.9	2.60	147.6

Symmetry codes: (i) 2/3-x, 1/3-x+y, 5/6-z; (ii) x-y,x, 1-z.
